# Supplementary material for: Adeno-associated virus delivered CXCL9 sensitizes glioblastoma to anti-PD-1 immune checkpoint blockade
Source: Nat Commun. 2024 Jul 12;15:5871. doi: 10.1038/s41467-024-49989-1 (PMC11245621; doi:10.1038/s41467-024-49989-1)
Supplement: Supplementary file 1 — Supplementary Information [file 41467_2024_49989_MOESM1_ESM.pdf]

Supplementary Figure 1. *AAV serotype screening in glioma*. (a) Reference IHC of CD3D expression in human GBM tumor tissue depicting undetectable and low staining frequencies (images from https://www.proteinatlas.org/ENSG00000167286-CD3D/pathology/glioma). Distribution of CD3D expression in human low grade glioma (LGG, n=7) and GBM (n=13), sorted into undetected, low (<25% positive cells), moderate (25-75% positive cells), or high (>75% positive cells) expression levels, based on the cumulative results of two independent antibody stains where low levels represent the highest degree of signal detected among all specimens examined. (b) Heatmap summary of EGFP fluorescence intensity detected *in vitro* in 15 primary human and murine glioma cell lines 72 hours following transduction with 29 unique AAV serotypes. (c) AAV6-EGFP control vector map. (d) AAV6-BFP control vector map. (e) AAV6-empty vector control map. (f) *In vitro* transduction efficiency of AAV6-EGFP detected via quantitative flow cytometry in GL261, KR158, and CT-2A murine glioma 72 hours post-transduction (1e5 VGS), n=3 per model. Statistical analyses were performed using two-way ANOVA with Sidak's multiple comparisons test. P-values = or < 0.05 are considered statistically significant. Bar graphs depict group mean with error bars representing standard deviation. Source data are provided as a Source Data File.

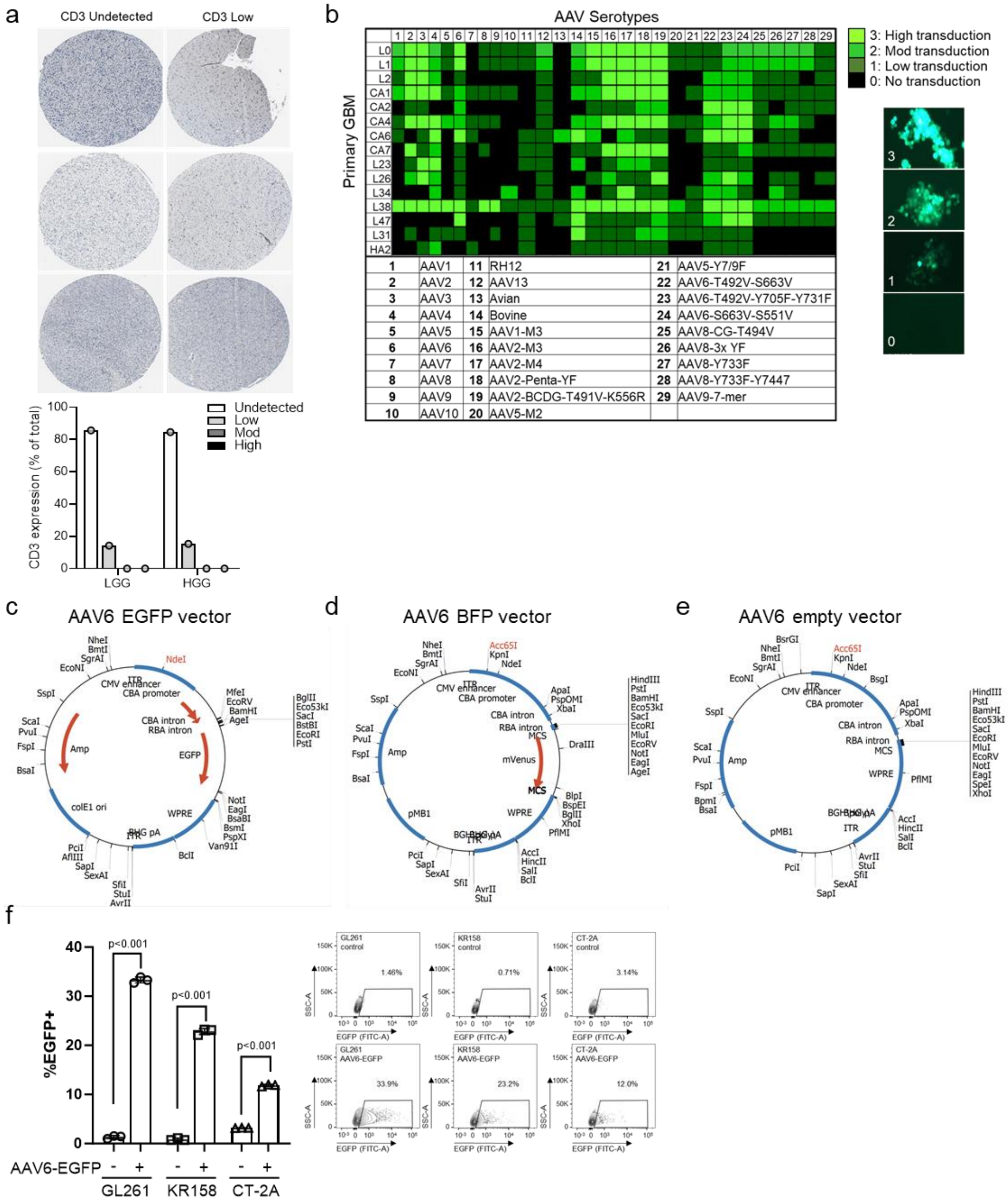

Supplementary Figure 2. *AAV6 3D biodistribution in tumor*. 3D IF of (a) KR158 and (b) GL261 tumors 1 week following AAV6-EGFP (green) intratumor injection (1e10 VGS) counter-labeled with DAPI nuclear stain (blue pseudocolor), n=3 per model. Left panels show 3D rendering, and the right panels depict select 2D images from Z-stack to show enhanced cellular resolution.

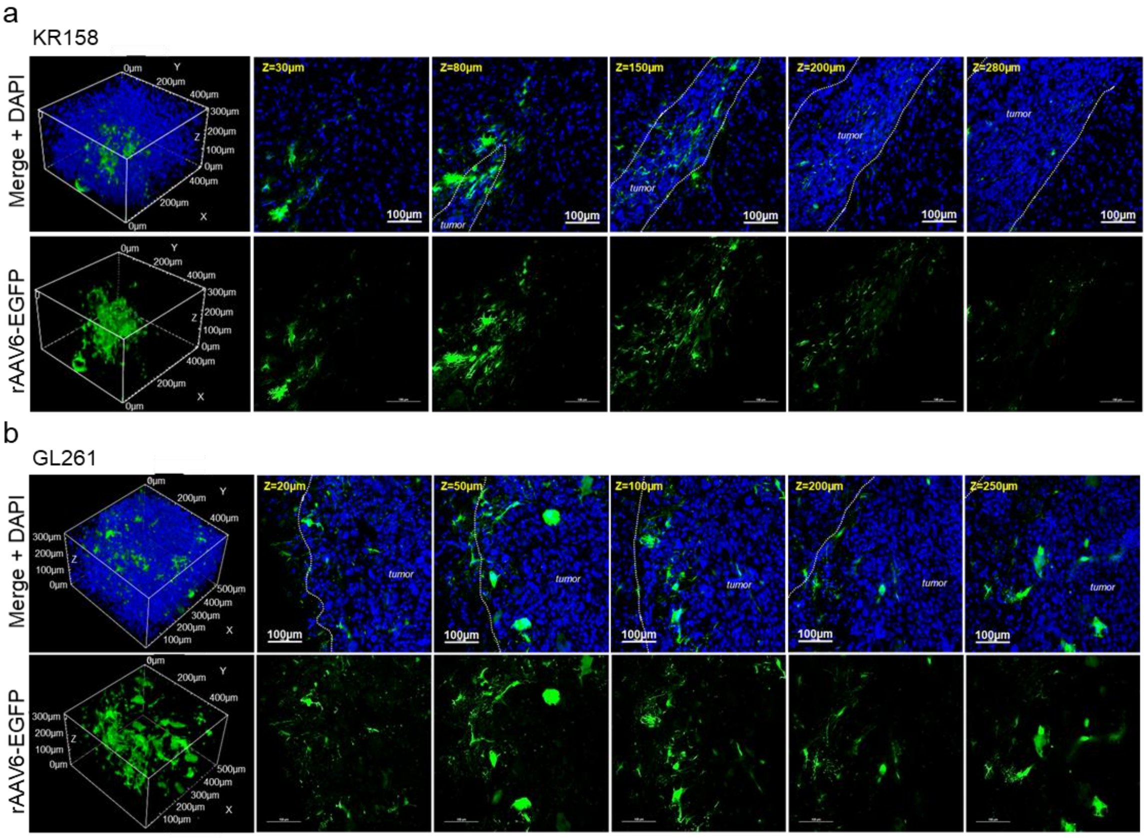

Supplementary Figure 3. *AAV6 co-localization analysis*. 3D IF of (a) KR158 and (b) GL261 tumors implanted in $CCR2^{RFP}CX3CR1^{GFP}$ reporter mice resected 1 week following intratumor injection with AAV6-BFP (1e10 VGS) and counter-labeled with DRAQ5 nuclear stain, n=2 per model. Left panels show 3D rendering (scale bar 50µm), and the right panels depict select 2D images from Z-stack to show enhanced cellular resolution. Digital zoom of selected regions outlined by yellow dashed line to show lack of overlap between BFP and either RFP (CCR2) and GFP (CX3CR1). (c) 3D IHC of RFP-labeled KR158 tumor tissue collected 1 week following AAV6-EGFP injection. The top row depicts 3D rendering of tissue captured at 10x magnification. AAV6 transduced cells are shown in green, GFAP in red, RFP+ tumor cells in gray, and DAPI nuclear stain in dark blue. 2nd and 3rd rows depict 2D digital zoom as outlined by the yellow dashed line in the top row to enhance cellular resolution. Voxel-based co-localization between AAV6 and GFAP (2nd row) and AAV6 and tumor cells (3rd row) is shown as a separate channel (yellow or pink). Representative images selected from n=4. (d) Quantitative summary of voxel-based AAV6 co-localization with either KR158 (n=4) or astrocytes (n=3) in tumor-bearing mice. Box-whisker plots display the box ranging from the first to the third quartile, the center median value, and the whiskers extend from each quartile to the minimum and maximum values. Source data are provided as a Source Data File.

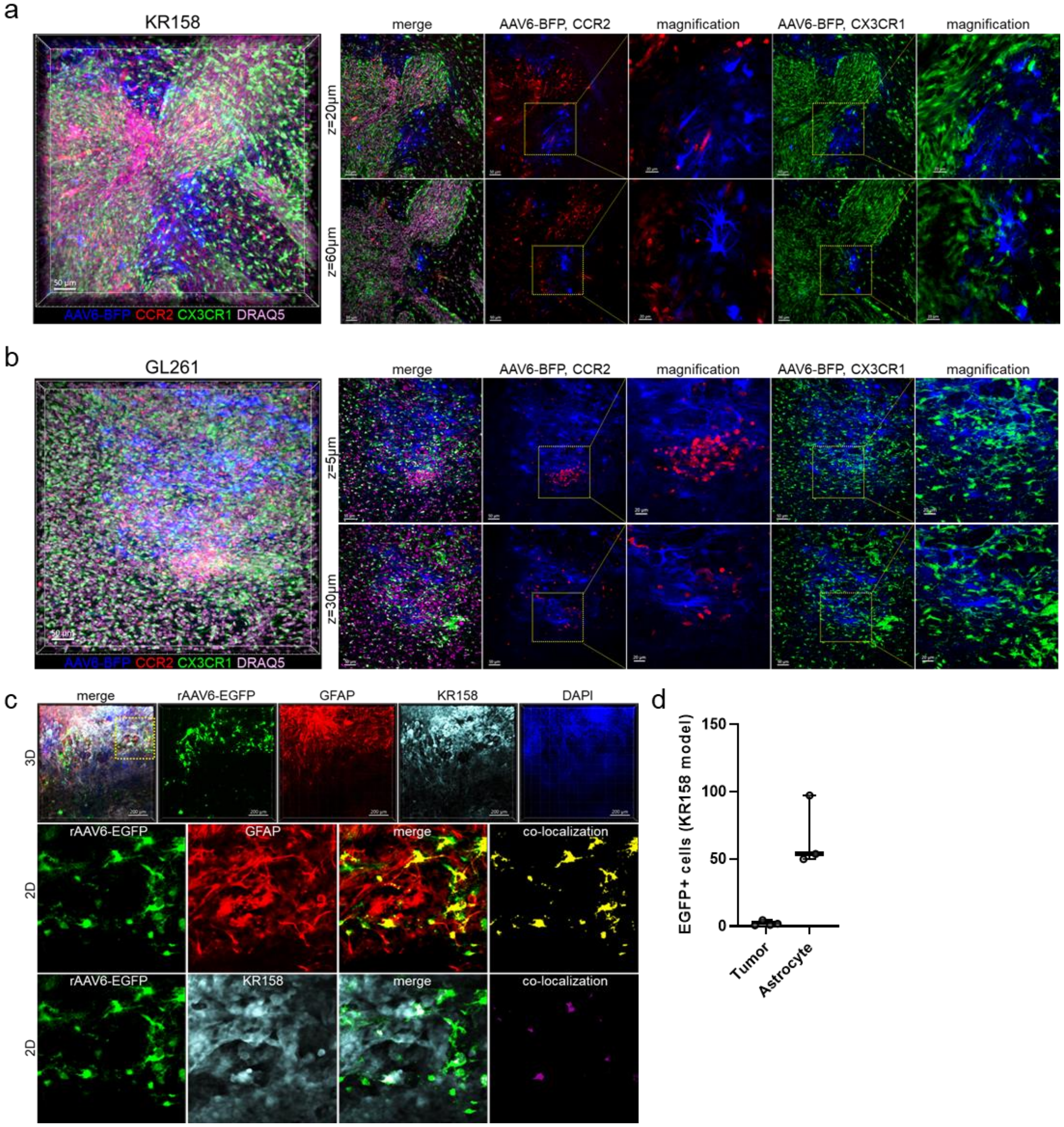

Supplementary Figure 4. *Flow cytometry gating strategies*. (a) Schematic illustration of the flow cytometry gating strategy and controls used in Fig. 1g. (b) Schematic illustration of the flow cytometry gating strategy and controls used in Fig. 3e-h, Fig. 4f, and Supplementary Fig. 5f-g.

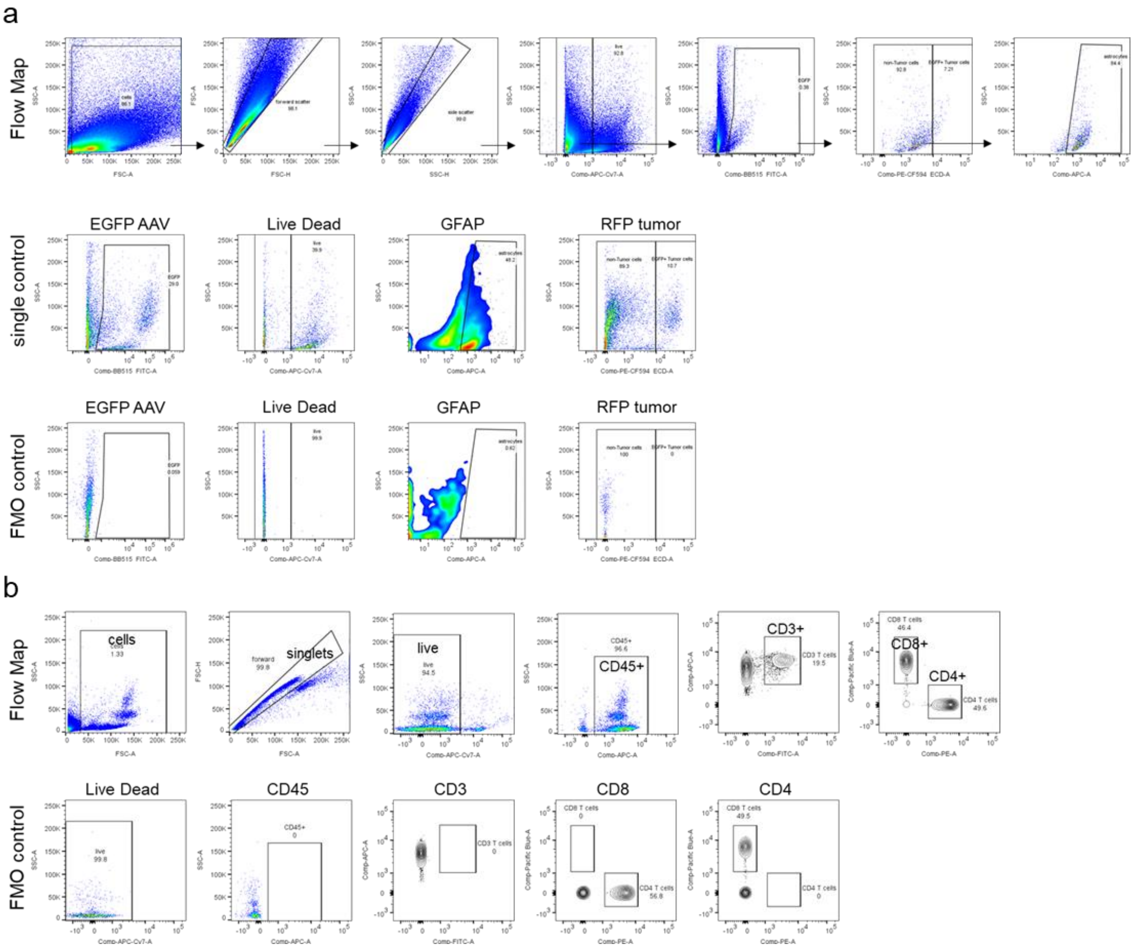

Supplementary Figure 5. *Mechanisms of therapeutic efficacy*. (a) Lymphocyte (CTV+, blue) chemotaxis in either AAV6-CXCL9 transduced GL261 field or AAV6-CXCL9 transduced C8-D1A field at 24-hours. Statistical analyses performed by Student's t-test, n=3. (b) ELISA detection of CXCL9 protein in media collected from AAV6-CXCL9 transduced C8-D1A and GL261 chambers immediately prior to co-culture with T lymphocytes. Statistical analyses performed by Student's t-test, n=3. (c) Proliferation analysis of C8-D1A and GL261 cells beginning 24h following AAV6-CXCL9 transduction. Data represents two independent tests, n=3 per group. (d) Flow cytometry detection of lymphocytes collected day 18 of CD8 depletion study. Bar graph depicts the percent of CD4 T lymphocytes (CD45+CD3+CD4+CD8-) and CD8 T lymphocytes (CD45+CD3+CD4-CD8+) detected within the total CD45+ population of PBMCs. Statistical analyses performed using Sidak's multiple comparisons test, n=5 Sham+IgG, n=7 Sham+CD8 depletion, n=8 AAV6-CXCL9+aPD-1 and AAV6-CXCL9+aPD-1+CD8 depletion. Individual values shown. (e) Flow cytometry detection of lymphocytes collected day 18 of CD4 depletion study. Bar graph depicting the percent of lymphocytes (CD45+CD3+CD4+CD8-) and CD8 T lymphocytes (CD45+CD3+CD4-CD8+) detected within the total CD45+ population of PBMCs. Statistical analyses performed using Sidak's multiple comparisons test, n=7 Sham+IgG, AAV6-CXCL9+aPD-1, and AAV6-CXCL9+aPD-1+CD4 depletion; n=6 Sham+CD4 depletion. Individual values shown. (f) Survival analysis in GL261 tumor-bearing mice treated with AAV6-CXCL9 delivered intratumorally (IT) or in the contralaterally (CL) in combination with anti-PD-1 ICB. Intratumor sham injected mice included as a control group, n=7 per group. Statistical analysis was performed using Log-rank (Mantel-Cox) test comparing individual treatment groups. (g) Survival analysis in GL261 tumor-bearing mice treated with AAV6-CXCL9 delivered intratumorally at day 5 or day 12 (delayed treatment, DT) following tumor implantation, in combination with anti-PD-1 ICB. Intratumor sham injected mice (day 5) included as a control group, n=7 sham+IgG and AAV6-CXCL9+aPD-1; n=6 delayed treatment. Statistical analysis performed using Log-rank (Mantel-Cox) test comparing individual treatment groups. (h) Representative whole brain 3D image (n=3) of a GL261-EGFP tumor (red) isolated 7 days following intratumor AAV-mCherry (green) delivery 12 days following tumor implantation, scale bar 500µm. Tissue immunolabeled for GFAP (cyan). P-values = or < 0.05 are considered statistically significant. Bar graphs depict group mean with error bars representing standard deviation. Source data provided as a Source Data File.

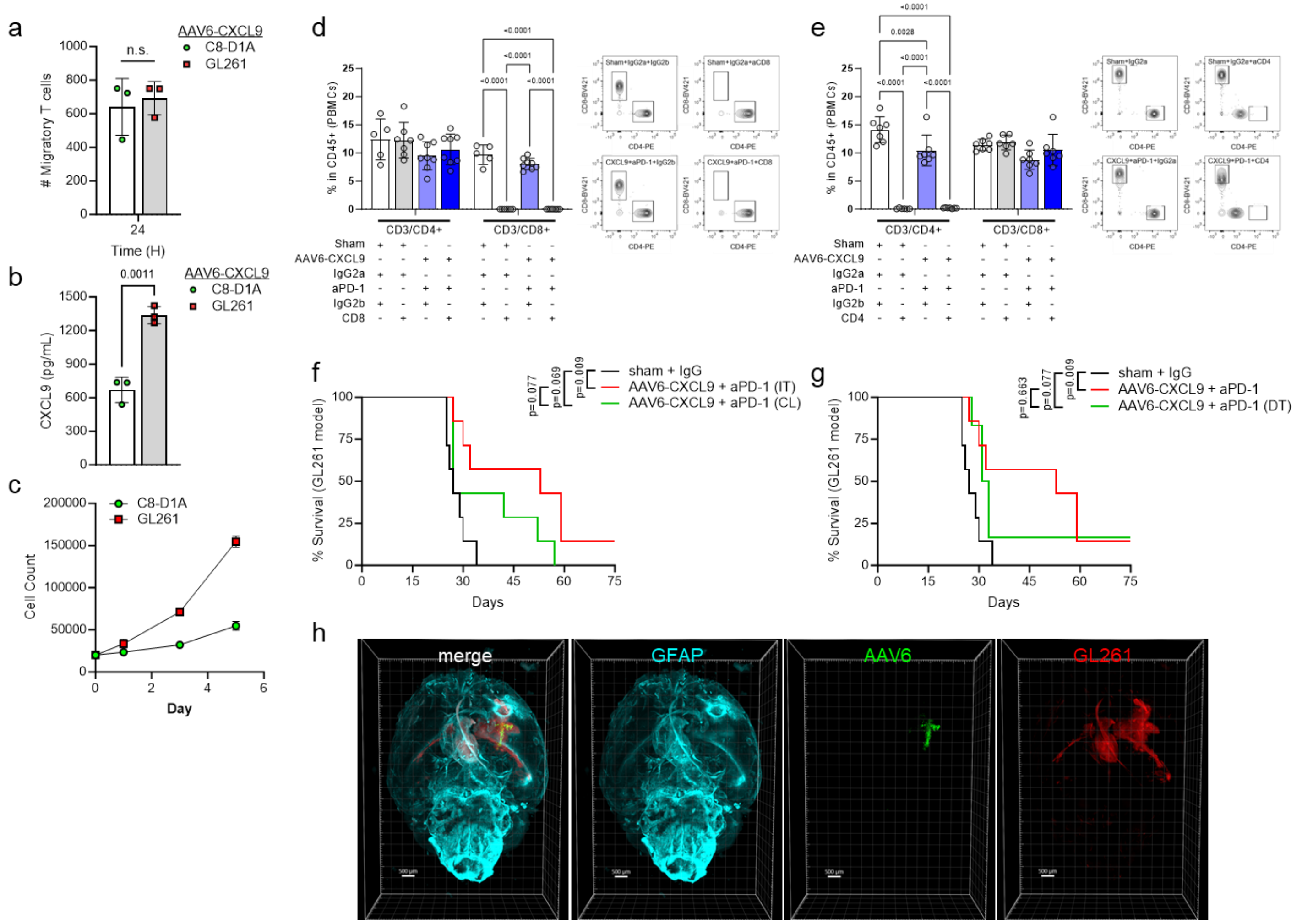

Supplementary Figure 6. *Total immune response to single and combination treatment*. Quantitative change in population frequency of (a) CD4+ T cells, (b) microglia, (c) dendritic cells (cDCs), (d) disease-associated microglia, (e) macrophages, (f) suppressive macrophages, (g) natural killer (NK) cells, and (h) border-associated macrophages (BAM) across treatment groups detected by scRNA-seq. Statistical analyses performed using ordinary one-way ANOVA with Fisher's least significant difference (LSD) test for multiple comparisons, n=3 per group, individual values shown. P-values = or < 0.05 are considered statistically significant. Box-whisker plots display the box ranging from the first to the third quartile, the center median value, and the whiskers extend from each quartile to the minimum and maximum values. P-values = or < 0.05 are considered statistically significant. Source data are provided as a Source Data File.

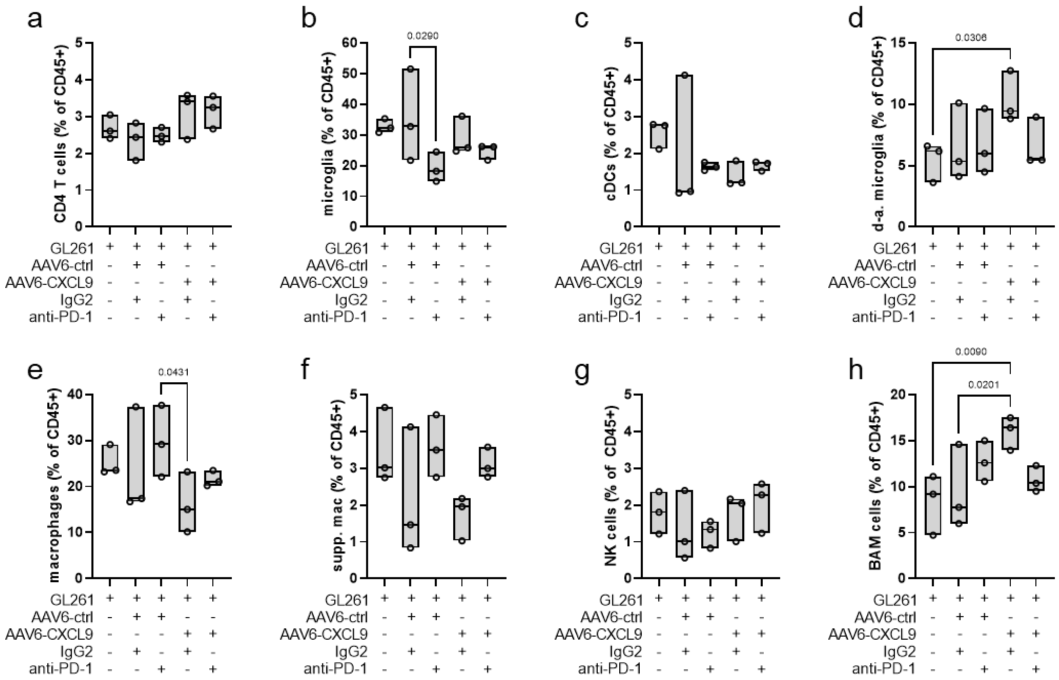

Supplementary Figure 7. *CXCR3 responsive lymphocytes*. (a) Summary of UMAP cell clusters. UMAP of cell types clustered by scRNA transcriptional analysis of 52,344 CD45+ cells isolated from GL261 tumor bearing mice treated with: (b) AAV6-ctrl + IgG2, (c) AAV6-ctrl + aPD-1, and (d) AAV6-CXCL9 + IgG2 treated GL261 tumors, n=3 mice per group. Summary circle chart depicting cell cluster population frequency detected for each treatment included alongside each UMAP. (e) UMAP display of *Cxcr3*, *Cd3e*, *Cd8a*, *Il7r*, *Foxp3*, *Klra7*, *Fcgr3*, *Tcf7*, *Gzmk*, *Itgae*, *Sell*, *Cd44*, *Pdcd1*, *Havcr2*, *Lag3,* and *Tigit* transcript expression used to define T cell clusters.

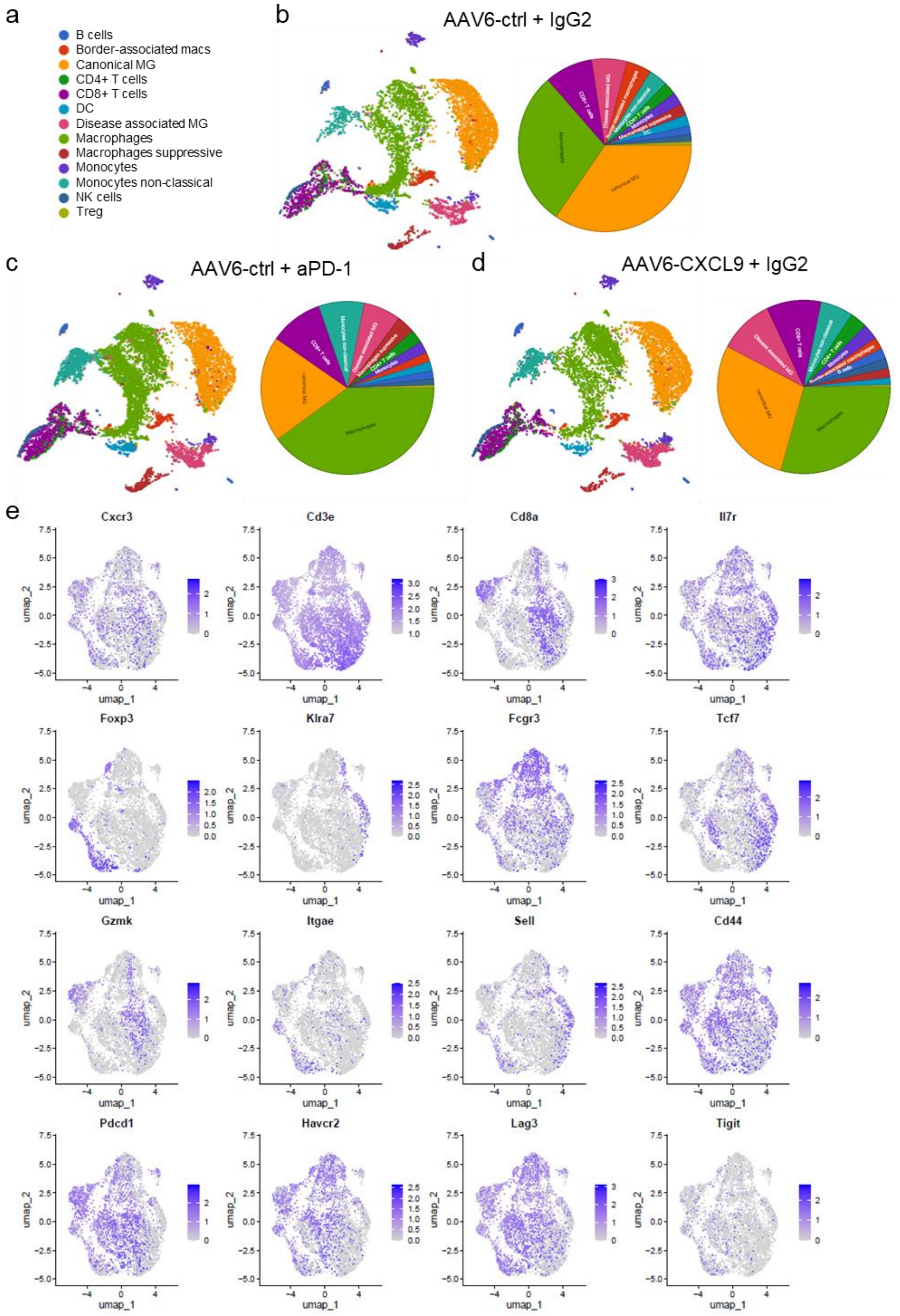

Supplementary Figure 8. *CD4 lymphocyte immune response to single and combination therapy*. Summary chord plots of ligand-receptor interactions between scRNA-seq defined cell clusters generated using CellChat[1], where line thickness correlates with the total number of predicted interactions between two defined cell clusters in each (a) sham, (b) AAV6-EGFP + IgG2, (c) AAV6-EGFP + aPD1, (d) AAV6-CXCL9 + IgG2, and (e) AAV6-CXCL9 + aPD1. (f) Waterfall summary plot of scRNA-seq-derived signaling pathways enriched in CD4+ T cells following combination AAV6-CXCL9 + aPD-1 as compared to AAV6-CXCL9 + IgG2 treatment. Data represents pooled interactions from n=3 mice per treatment group. (g) Waterfall summary plot of scRNA-seq-derived signaling pathways enriched in CD4+ T cells following combination AAV6-CXCL9 + aPD-1 as compared to AAV6-EGFP + aPD-1 treatment. (h) Summary of pathway enrichment analysis for immune cells performed with AUCell algorithm using the nanoString nCounter® Immune Exhaustion pathway dataset. Statistical analysis was performed using Kruskal-Wallis and Dunn's multiple comparison tests. P-values = or < 0.05 are considered statistically significant. Box-whisker plots display the box ranging from the first to the third quartile, the center median value, and the whiskers extend from each quartile to the minimum and maximum values.

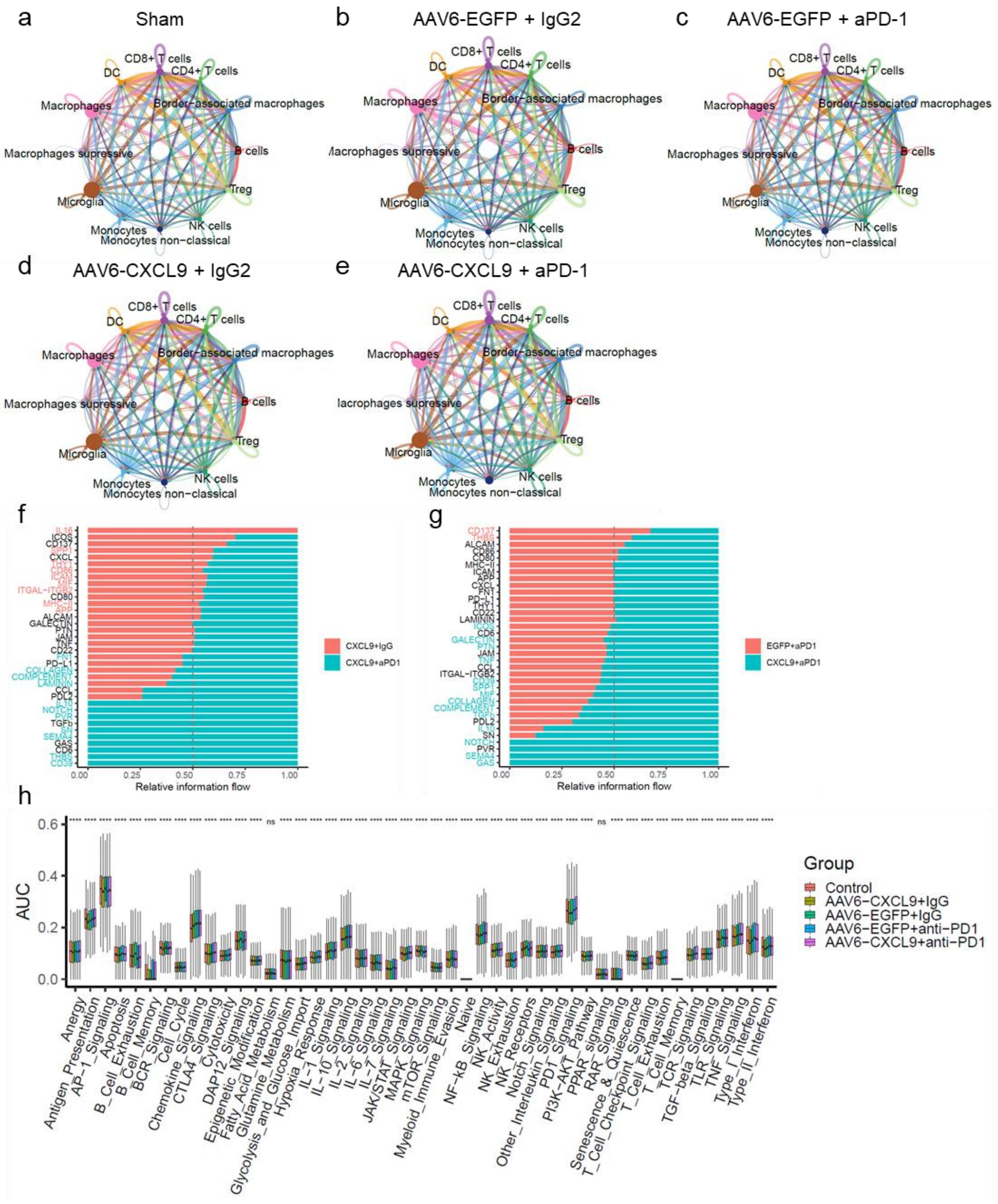

Supplementary Figure 9. *Monocyte immune response to single and combination therapy*. (a) Heatmap representation of gene expression analysis derived from monocytes using the nCounter® Immune Exhaustion Panel (nanoString) following AAV6-CXCL9 gene therapy with or without PD-1 ICB. (b-m) Quantification of common pathways differentially expressed in monocytes in response to treatment, derived from n=669, 674, 1099, 912, and 1656 single cells pooled from n=3 individual samples per treatment group, graphically presented from left to right. Statistical analyses performed using Kruskal-Wallis test followed by Dunn's multiple comparisons, with individual values shown. P-values = or < 0.05 are considered statistically significant. Box-whisker plots display the box ranging from the first to the third quartile, the center median value, and the whiskers extend from each quartile to the minimum and maximum values. Source data are provided as a Source Data File.

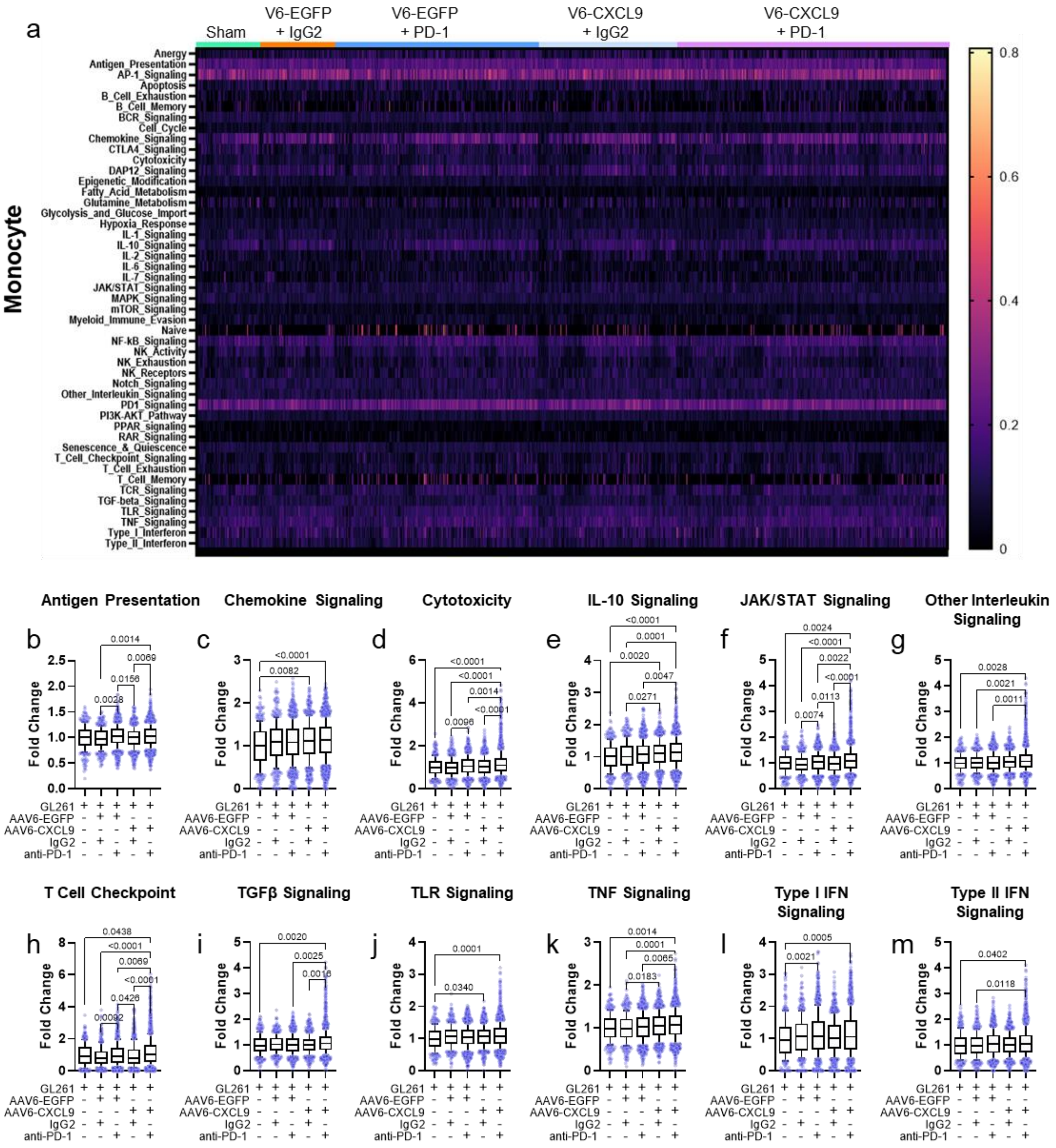

Supplementary Figure 10. *Proteomic analysis of immune response to single and combination therapy*. (a) Heatmap of relative protein expression of 65 out of 111 total inflammatory cytokines detected in sham control, single agent, and combination AAV6-CXCL9 plus anti-PD-1 checkpoint inhibitor treated tumor tissue collected 10 days after the onset of treatment, n=3-4 per group. Undetected proteins not displayed. (b-k) Violin plots of relative protein expression of differentially expressed chemokines and cytokines detected in tumors in response to AAV6-CXCL9 with or without anti-PD-1 ICB. Statistical analyses performed using Kruskal-Wallis multiple comparisons test, individual values shown (n=3 for sham, AAV6-EGFP + IgG2a, and AAV6-EGFP + aPD-1; n=4 for AAV6-CXCL9 + IgG2a and AAV6-CXCL9 + aPD-1 for all panels). P-values = or < 0.05 are considered statistically significant. Source data are provided as a Source Data File.

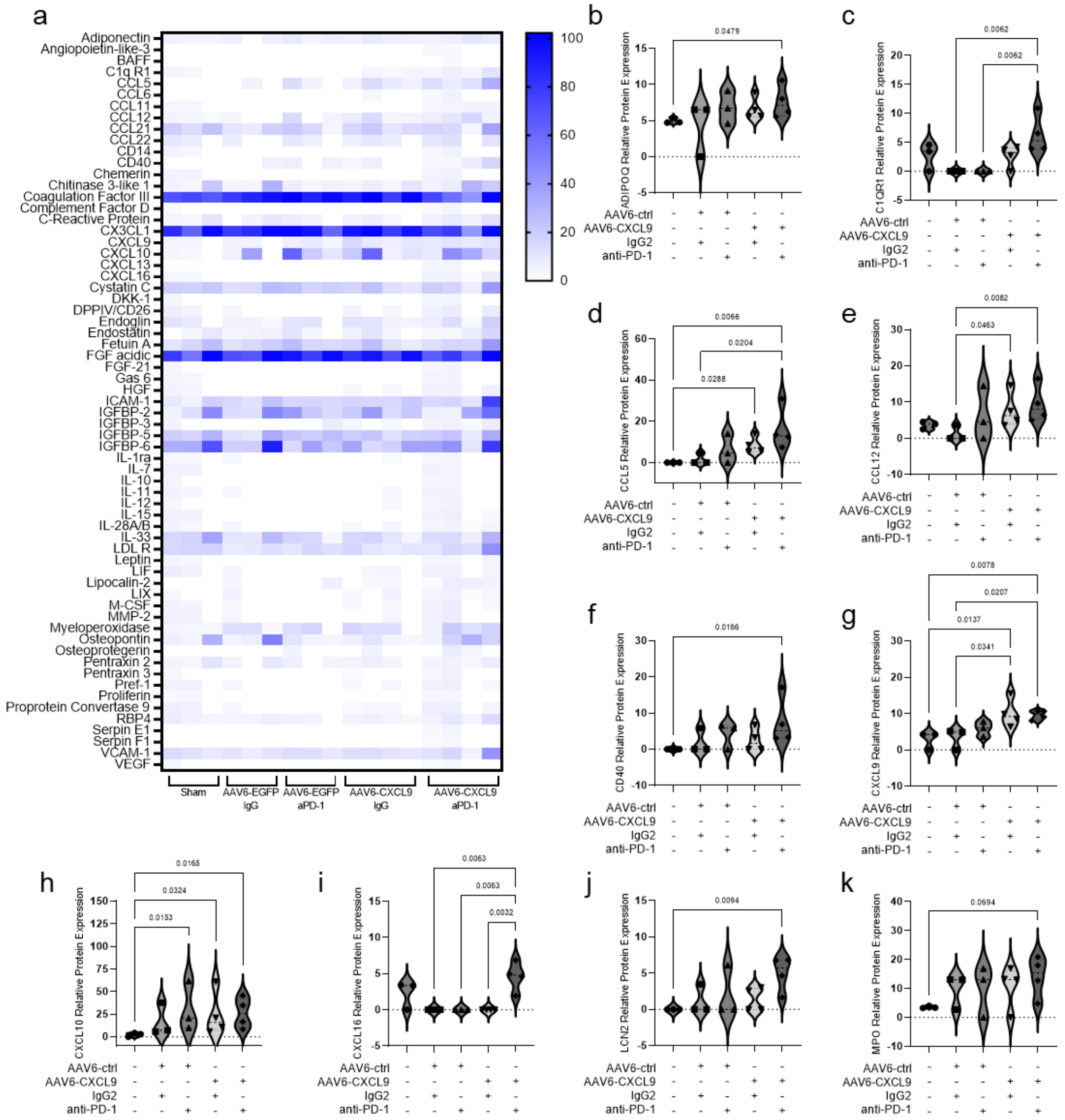

Supplementary Table 1. *Summary of known marker genes used to define scRNA-seq unique cell clusters.*

Target group	Gene markers			Reference
canonical MG	Fcrls	Tmem119	P2ry12	Ochocka et al [2]
Disease associated MG	Lpl	Cst7		Ochocka et al [2]
Macrophages	Ly6c2	Ccr2	Ifitm2	Ochocka et al [2]
Border-associated macrophages	Cd163	Mrc1	Lyve1	Ochocka et al [2]
Macrophages suppresive	Ccl22	Cd274		Ochocka et a l[2]
Monocytes	Ly6c1			Ochocka et al [2]
Monocytes non-classical	Spn	Cx3cr1	Tnfrsf1b	Ochocka et al [2]
DC	Xcr1	Flt3	Cd24a	Hongo et al [3]
NK cells	Klra1	Ncr1		Ochocka et al [2]
B cells	Cd79a	Cd79b	Ms4a1	Lee et al [4]
CD8+ T cells	Cd3d	Cd8a	Cd8b1	Zheng et al [5]
CD4+ T cells	Cd3d	Cd4		Zheng et al [5]
Treg	Cd4	Foxp3	Il2ra	Zheng et al [5]

Supplementary References

1. Jin, S., *et al*. Inference and analysis of cell-cell communication using CellChat. *Nat Commun* **12**, 1088 (2021).
2. Ochocka, N., *et al*. Single-cell RNA sequencing reveals functional heterogeneity of glioma-associated brain macrophages. *Nat Commun* **12**, 1151 (2021).
3. Hongo, D., *et al*. Identification of Two Subsets of Murine DC1 Dendritic Cells That Differ by Surface Phenotype, Gene Expression, and Function. *Front Immunol* **12**, 746469 (2021).
4. Lee, R.D., *et al*. Single-cell analysis identifies dynamic gene expression networks that govern B cell development and transformation. *Nat Commun* **12**, 6843 (2021).
5. Zheng, G.X., *et al*. Massively parallel digital transcriptional profiling of single cells. *Nat Commun* **8**, 14049 (2017).
