# Supplementary material for: Adeno-associated virus delivered CXCL9 sensitizes glioblastoma to anti-PD-1 immune checkpoint blockade
Source: Nat Commun. 2024 Jul 12;15:5871. doi: 10.1038/s41467-024-49989-1 (PMC11245621; doi:10.1038/s41467-024-49989-1)
Supplement: Supplementary file 3 — Reporting Summary [file 41467_2024_49989_MOESM3_ESM.pdf]

nature portfolio

Corresponding author(s): Christina von Roemeling, Duane Mitchell

Last updated by author(s): Apr 12, 2024

Reporting Summary

Nature Portfolio wishes to improve the reproducibility of the work that we publish. This form provides structure for consistency and transparency in reporting. For further information on Nature Portfolio policies, see our Editorial Policies and the Editorial Policy Checklist.

Statistics

For all statistical analyses, confirm that the following items are present in the figure legend, table legend, main text, or Methods section.

n/a	Confirmed	
☐	☒	The exact sample size (*n*) for each experimental group/condition, given as a discrete number and unit of measurement
☐	☒	A statement on whether measurements were taken from distinct samples or whether the same sample was measured repeatedly
☐	☒	The statistical test(s) used AND whether they are one- or two-sided<br>*Only common tests should be described solely by name; describe more complex techniques in the Methods section.*
☒	☐	A description of all covariates tested
☐	☒	A description of any assumptions or corrections, such as tests of normality and adjustment for multiple comparisons
☐	☒	A full description of the statistical parameters including central tendency (e.g. means) or other basic estimates (e.g. regression coefficient) AND variation (e.g. standard deviation) or associated estimates of uncertainty (e.g. confidence intervals)
☐	☒	For null hypothesis testing, the test statistic (e.g. *F*, *t*, *r*) with confidence intervals, effect sizes, degrees of freedom and *P* value noted<br>*Give P values as exact values whenever suitable.*
☒	☐	For Bayesian analysis, information on the choice of priors and Markov chain Monte Carlo settings
☒	☐	For hierarchical and complex designs, identification of the appropriate level for tests and full reporting of outcomes
☒	☐	Estimates of effect sizes (e.g. Cohen's *d*, Pearson's *r*), indicating how they were calculated

Our web collection on statistics for biologists contains articles on many of the points above.

Software and code

Policy information about availability of computer code

Data collection	GraphPad Prism version 10, Microsoft Excel 2016, Microsoft PowerPoint 2016, Nikon NIS-Elements, Imaris x64 version 9.7.0, FlowJo version 10.8.1
Data analysis	Cell Ranger software 10x Genomics (version 7), Partek Flow analysis software (version 10), AUCell algorithm using the NanoString nCounter Immune Exhaustion panel, CellChat algorithm, pheatmap package (version 1.0.12), Circos visualization software, FlowJo software (version 10.8.1), Imaris x64 (version 9.7.0), GraphPad Prism (version 10).

For manuscripts utilizing custom algorithms or software that are central to the research but not yet described in published literature, software must be made available to editors and reviewers. We strongly encourage code deposition in a community repository (e.g. GitHub). See the Nature Portfolio guidelines for submitting code & software for further information.

Data

Policy information about availability of data

All manuscripts must include a data availability statement. This statement should provide the following information, where applicable:

- Accession codes, unique identifiers, or web links for publicly available datasets
- A description of any restrictions on data availability
- For clinical datasets or third party data, please ensure that the statement adheres to our policy

The data that support the findings of this study are available from the corresponding author upon reasonable request. All equipment and reagents are commercially available and are described in the Methods section. scRNAseq data files were uploaded into the open-access Genome Sequence Archive database (OMIX005735).

Research involving human participants, their data, or biological material

Policy information about studies with human participants or human data. See also policy information about sex, gender (identity/presentation), and sexual orientation and race, ethnicity and racism.

Reporting on sex and gender	not applicable
Reporting on race, ethnicity, or other socially relevant groupings	not applicable
Population characteristics	not applicable
Recruitment	not applicable
Ethics oversight	not applicable

Note that full information on the approval of the study protocol must also be provided in the manuscript.

Field-specific reporting

Please select the one below that is the best fit for your research. If you are not sure, read the appropriate sections before making your selection.

☒ Life sciences ☐ Behavioural & social sciences ☐ Ecological, evolutionary & environmental sciences

For a reference copy of the document with all sections, see nature.com/documents/nr-reporting-summary-flat.pdf

Life sciences study design

All studies must disclose on these points even when the disclosure is negative.

Sample size	No effect size was predetermined. The sample sizes were kept consistent with previously published study where in vitro studies were repeated at least three times independently. For qualitative in vivo studies (e.g. AAV transduction co-localization) a minimum of n=3 per group was used. For in vivo survival experiments, a minimum of 5 mice per group was used, and was estimated to achieve the detection of significant differences between groups based on means and standard deviations. Details regarding the sample size of all experiments are provided in the figure legends.
Data exclusions	No data were excluded from final analyses.
Replication	A minimum of three replicates were performed for each experiment. Graphical data presents all individual values from pooled datasets along with data distribution or mean +/- standard deviation.
Randomization	For in vivo studies, animals were randomized following tumor implantation before treatment was initiated.
Blinding	The investigators and authors have been consistently blinded to the group allocation during data collection and analysis.

Reporting for specific materials, systems and methods

We require information from authors about some types of materials, experimental systems and methods used in many studies. Here, indicate whether each material, system or method listed is relevant to your study. If you are not sure if a list item applies to your research, read the appropriate section before selecting a response.

Materials & experimental systems

n/a	Involved in the study	
	x	Antibodies
	x	Eukaryotic cell lines
x		Palaeontology and archaeology
	x	Animals and other organisms
x		Clinical data
x		Dual use research of concern
x		Plants

Methods

n/a	Involved in the study	
x		ChIP-seq
	x	Flow cytometry
x		MRI-based neuroimaging

Antibodies

Antibodies used

Flow cytometry antibodies:
CD45-APC (Biolegend, cat# 103112, 0.20 ug per 10^6 cells)
CD3-FITC (Biolegend, cat# 100204, 1.0 ug per 10^6 cells)
CD4-PE (Biolegend, cat# 100408, 0.20 ug per 10^6 cells)
CD8-BV421 (Biolegend, cat# 100738, 0.50 ug per 10^6 cells)
GFAP-APC (ThermoFisher, cat# 51-9792-82, 0.50 ug per 10^6 cells)

Immunolabeling of tissue:
GFAP (Thermo Fisher, cat# PA1-10004, 1:50 dilution)
CD45 (Thermo Fisher, cat# 14-0451-82, 1:50 dilution)
anti-chicken Alexa FluorTM 647 antibody (Thermo Fisher, cat# A-21449, 1:200 dilution)
anti-rat Alexa FluorTM 568 (Thermo Fisher, cat# A-11077, 1:200 dilution)

Validation

All antibodies are commercially available and have been validated by the manufacturer.

Eukaryotic cell lines

Policy information about cell lines and Sex and Gender in Research

Cell line source(s)

L0, L1, L2, CA1, CA2, CA4, CA6, CA7, L23, L26, L31, L34, L38, L47, and HA2 were a kind gift from Dr. Brent A. Reynolds (University of Florida) and were generated directly from patient clinical specimens. KR158B-luc (Kluc) glioma line was provided by Dr. Karlyne M. Reilly (NCI Rare Tumor Initiative, NIH) and GL261 obtained from the DCTD Tumor Repository (NCI). CT-2A were purchased from Millipore Sigma. C8-D1A primary astrocytes were purchased from ATCC.

Authentication

Gene expression analysis of Kluc and GL261 confirmed appropriate haplotype background and expression of astrocytoma-associated genes. CT-2A was verified by Millipore Sigma using STR-PCR. C8-D1A was verified by ATCC using STR-PCR.

Mycoplasma contamination

All cell lines were tested negative for mycoplasma contamination.

Commonly misidentified lines
(See ICLAC register)

No misidentified cell lines used in the study.

Animals and other research organisms

Policy information about studies involving animals; ARRIVE guidelines recommended for reporting animal research, and Sex and Gender in Research

Laboratory animals

C57BL/6J (Strain# 000664), CCR2RFPCX3CR1GFP (Strain# 032127), GREAT (Strain# 017581), and UBC-GFP (Strain# 004353) mice (Six- to eight-week-old, female) were purchased from Jackson Laboratory and maintained at the animal facility of the University of Florida in ventilated cages in a pathogen-free facility in a standard environmentally controlled room, with 50% humidity and 22C temperature under a 14-10h light-dark cycle. Standard water and diet were given to the mice.

Wild animals

not applicable

Reporting on sex

female

Field-collected samples

not applicable

Ethics oversight

This research complies with all relevant ethical regulations. Protocols were reviewed and approved by the University of Florida Institutional Animal Care and Use Committee.

Note that full information on the approval of the study protocol must also be provided in the manuscript.

Plants

Seed stocks	not applicable
Novel plant genotypes	not applicable
Authentication	not applicable

Flow Cytometry

Plots

Confirm that:

- [x] The axis labels state the marker and fluorochrome used (e.g. CD4-FITC).
- [x] The axis scales are clearly visible. Include numbers along axes only for bottom left plot of group (a 'group' is an analysis of identical markers).
- [x] All plots are contour plots with outliers or pseudocolor plots.
- [x] A numerical value for number of cells or percentage (with statistics) is provided.

Methodology

Sample preparation	Brain tissue was digested using the Multi-tissue Dissociation Kit (Miltenyi Biotec) on a gentleMACS Octo Dissociator with heat, followed by sample clean-up using Debris Removal Solution (Miltenyi Biotec) according to manufacturer's protocol. Tumor-infiltrating leukocytes were isolated using CD45 microbeads (Miltenyi Biotec) filtered through LS columns (Miltenyi Biotec) on a QuadroMACS Separator (Miltenyi Biotec) according to manufacturer's protocol. Blood samples were collected from the anterior vena cava, and RBC lysis performed using Pharm Lyse solution (BD Biosciences) per manufacturer's protocol. Samples were washed 2x with cold PBS. Unstained cells were reserved for unlabeled and FC controls, and dead cells were labeled with Zombie NIRTM Fixable Viability Kit (Biolegend) according to manufacturer's protocol. Cells were washed 2x in PBS containing 0.5% BSA (Sigma) and 2mM EDTA (Thermo Fisher) FC buffer and blocked for 10 minutes on ice using TruStain FcX (Biolegend) prior to cell surface antigen labeling with the antibodies listed above.For astrocyte detection, cells were fixed and permeabilized using True-Nuclear Transcription Factor Buffer Set (Biolegend) following manufacturer's protocol following debris removal step, with no CD45 microbead isolation. Following immunolabeling, all samples were washed 2x with FC buffer and analyzed using a BD FACSymphony A3 flow cytometer.
Instrument	BD FACSymphony A3 flow cytometer
Software	FlowJo software (version 10.8.1)
Cell population abundance	No sorting was performed
Gating strategy	All gating strategies and fluorescence-minus-one (FMO) controls are provided in the supplementary figures. The negative population was determined by using unstained cells and FMO controls. The population with fluorescence intensity higher than that of FMO controls is considered positive and the other population is considered negative.

- [x] Tick this box to confirm that a figure exemplifying the gating strategy is provided in the Supplementary Information.
